# Mr. Howship, on the Diseases of the Urinary Organs

**Published:** 1816-11

**Authors:** 


### *Mr. Howship, on the Diseases of the Urinary Organs.*

( Continued from page 290 )

CHAP. 5. *Diseases of the Prostate Gland.* Temporary affections of this body from strictures, gonorrhœa, &c. occur, but not frequently till after 60. When from repelled gonorrhœal matter, the neck of the bladder and prostate gland become affected, an uneasy sense of weight is felt at the root of the urethra; and on examination *per anum*, the gland will feel full, tumid, and rather tender. This tenderness will also be felt in voiding fæces. One of the lateral parts, or the small posterior lobe, generally takes the lead, and is found enlarged, or morbidly altered in structure from the rest of the gland. The process is, in general, slow; the action chronic; and in itself not painful.

*Symptoms.* Some degree of impediment in making water, gradually increasing till strangury takes place; a ropy tenacious secretion in the urine, offensive in odour, and soon putrifying; great straining, as the enlargement increases, ending sometimes in violent inflammation of the mucous membrane of the bladder, or even the muscular; terminating usually in a consolidation of the general structure, incapable of future relaxation. In cases of enlarged prostate, or obstruction of the urethra, the secretion of urine is actually diminished; sometimes with distention of the kidnies, sometimes with complete non-secretion; the patient dying comatose. When complicated with stricture, a careful investigation is necessary, to ascertain whether the disease of the prostate be primary or secondary. Examination by the finger is here useful.

*Treatment, in the early stages.* Where prostate enlargement arises from urethral stricture, there is little to apprehend; the tumour is formed in the lateral parts, not the middle lobe, and is removed with the stricture. The prostatal affection from gonorrhœa is transitory, and relieved by the increased secretion from the mucous membrane at the neck of the bladder. In this case the irritation is to be restored as quickly as possible to its proper seat, the anterior extremity of the urethra by the warm bath, fomentations, and, if necessary, the application of some irritating substance as the bals. copaib. by means of a bougie. The regular introduction of the latter will also be indispensable. The parts may, in some cases, be still further relieved by the abstraction of blood locally; and with this view, some leeches may be applied to the perinæum, or the patient may be cupped upon the loins. Where, however, frequency and uneasiness in making water appear, for the first time, towards the decline of life, there will be good reason to believe that the prostate gland is affected. In the early progress of the complaint, it admits of considerable relief. The means are, perfect tranquillity; abstraction of blood from the loins or perinæum; open bowels; abstinence from wine and spirits; low regimen; the hip bath; and an opiate at night. In this stage the introduction of a bougie or catheter would obviously be injurious.

*Introduction of the Catheter.* When circumstances render this operation necessary, Mr. H. prefers the flexible gum catheter to the silver one, if it can be introduced; with or without the stilet.

"In those instances where the introduction of the flexible catheter has been effected with difficulty, it will be necessary that it be allowed to remain in the bladder for a certain number of days, for the purpose of insuring the bladder being relieved at regular and stated intervals, either twice or thrice in the 24 hours." 155.

*False passages.* Mr. Howship justly observes, that these are often formed by the attempts of patients to pass bougies on themselves.

"I was lately requested, says our author, to examine the body of a physician, who, for many years, was one of the most approved teachers in London in his particular department of medical science. He had been for many years subject to strictures in the urethra, and had been long in the habit of passing bougies for himself. In consequence of this practice, he had formed two false passages; one of these was anterior to the verumontanum, and passed obliquely out of the urethra, for the extent of ½ of an inch, forming a *cul de sac;* the other was situated higher up, passing between the substance of the prostate gland and the inner membrane lining the canal, to the extent of ⅝ of an inch, where it again opened into the cavity of the bladder." 157.

In some cases of urgency, where the degree of prostatal enlargement prevented the possibility of introducing the silver catheter by gentle means, force has been used, in a few instances with success; a practicable passage remaining for the future introduction of the catheter, without the patient appearing to suffer any ill consequences.

"I know of one instance, however, in which a very excellent anatomist, who is since dead, in performing this operation, at the earnest entreaty of the patient, unfortunately passed the catheter through the body of a very vascular and irritable fungus that had sprung up internally from the surface of the diseased gland. The patient was instantly thrown into the most excruciating agony, and survived only half an hour."

In another instance, where the catheter was pushed through the diseased prostate, hæmorrhage took place; formed a coagulum in the bladder, and the patient died.

When the enlargement or excitement of the prostate is considerable, a great secretion takes place, forming a transparent colourless deposit at the bottom of the vessel, which, when stirred up, will be found as ropy and tenacious as the white of an egg. This increased secretion from the gland, though it implies no specific state of disease, merits attention, as serving to identify the particular seat of the affection. When the prostatal enlargement has gone so far as to produce considerable difficulty in expelling the urine, it is apt to derange the functions of the kidnies, and diminish urinary secretion. Hence the necessity of drawing off the water at stated intervals, twice or oftener a day, as the only means by which the activity of the circulation through the kidnies can be restored.

We shall pass over several interesting *cases* of diseased prostate, elucidatory of the foregoing observations, and for which we refer to the volume itself We now come to an important subject—urethral stricture, which appears to have occupied much of Mr. Howship's attention, and to have elicited much acuteness of remark. From Mr. H's. section on the stricture of the urethra, we shall select a passage relative to the dispute about its muscularity.

"But it is under circumstances of indisposition, that the strongest evidence is afforded of the membrane of the urethra possessing a muscular power. Indeed, the fact may be proved almost in any instance, by introducing a bougie of moderate size into th[illegible]

healthy urethra, and lightly supporting the end that projects from the penis, in a horizontal position. If the action of the urethra is then watched with attention, it will be found, that the power which expels the instrument; in other words, the contraction of the urethra, is uniform through its whole extent. The point of the bougie is not pushed forward more quickly while it moves through the bulb of the urethra, where the canal is surrounded with strong muscles, than it is afterward; but on the contrary, its motion is exceeding slow and perfectly equal throughout, until the whole of the instrument is expelled, and the point fairly drops from the orifice of the urethra. Late authors may say, this is clearly the elasticity of the living membrane; and perhaps had the bougie been passed only to the extent of half an inch into the urethra, its expulsion upon this principle might admit of doubt; but when the instrument has been introduced several inches, the advocates for elasticity forget that while the membrane of the urethra is exertingitself, for one eighth of an inch, to push forward the end of the bougie, the same power is operating against it to the superior extent of several inches, and that the considerable friction from so extensive a surface of contact must prevent the possibility of elasticity having any thing to do in the expulsion of the instrument." 181.

*Spasmodic Stricture.* There are many grounds for belief in spasmodic stricture. First, the causes which produce contraction in the urethra are the same as those which effect irritation in any other muscular part. Secondly, the suddenness with which the effect follows the cause, affords a strong argument in favour of pure spasm. Lastly, it is proved by the immediate relief afforded by antispasmodics.

But as this part of the urethra necessarily becomes the most irritable part of the canal, any change in diet, excess in wine, exercise or fatigue, may be sufficient to aggravate the degree of spasm, excite inflammation, and even bring on complete retention of urine; hence, spasmodic affection becomes gradually converted into organic or permanent contraction. Where the stricture has been induced by sympathy with some of the surrounding parts, as in disease of the rectum, bladder, or prostate, the stricture will be at first purely spasmodic; and likely to remain such. But when an irritating cause has been brought at once into contact with the inner membrane of the urethra, as astringent injections in gonorrhœa, or calculus arrested in its passage through the urethra, exciting extreme pain and violent inflammation, then effusion of coagulable lymph takes place into the surrounding cellular texture, laying the foundation for the most obstinate and extensive strictures. Mr. H. is decidedly of opinion, that injections, "through the medium of inflammation, not only leave stricture, but stricture of the worst and most obstinate kind"; in which opinion we coincide from pretty extensive experience in this complaint. External violence may operate in producing stricture through irritation, inflammation, or ulceration. Blisters have sometimes produced temporary stricture.

Next to the membranous part, the bulb of the urethra is the most frequent seat of stricture; as being the most irritable portion of the canal. The symptoms of stricture, though so extremely insidious in their approach, as generally to elude the patient's notice till considerable progress is made, are yet so familiar to the practitioner, that we shall pass them over.

*Treatment.* The spasmodic stricture is occasionally relieved by opiates, but "the principal means is the use of the bougie."

"With regard to the comparative merits of the different kinds of bougies, there have been various opinions. The common wax bougie is that which is in most general use, and for several reasons I think it deserves the preference it has obtained among practitioners. Some surgeons are in the habit of using bougies formed of catgut; and in very contracted strictures that will not allow the smallest sized wax bougie to enter, the catgut bougie often proves useful, and may be tried with advantage. But it has been objected to the common bougie that it loses its proper consistence when allowed to remain in the urethra any length of time; and this objection applies in a much greater degiee to the bougie of catgut, which absorbs moisture rapidly, swelling, and untwisting its fibres to that degree as to occasion considerable irritation at the neck of the bladder, and giving great pain when it is withdrawn.

"The bougie of elastic gum is certainly less liable to these inconveniences than either the catgut, or the common bougie; and it appears to me, that in cases where stricture is connected with affection of the prostate gland, the gum elastic may be more usefully employed than the wax bougie; at least I have found it upon trial in two or three such instances answer much better, passing through the stricture with more ease, and slipping over the projecting parts of the enlarged prostate gland with much less uneasiness to the patient, and without exciting any of that irritation which would have rendered the introduction of the common bougie improper; a circumstance that can be attributable only to the superior softness which enables the elastic gum even to follow with facility the course of the canal.

"One quality considered desirable in a bougie, is a power of receiving and retaining any particular degree of curvature that may be chosen; and upon this ground principally has the metallic bougie been introduced into practice. This bougie, upon first view, might be expected to answer very well, but it is, notwithstanding, in my opinion, a most objectionable instrument. There have been several instances in which these bougies have broken in the urethra, a part has escaped into the bladder, and it has been found necessary to save the patient from the ill consequences of irritation and inflammation of the bladder, by cutting it out, as in the operation for the stone." 202.

In the mode of applying the bougie, many judicious directions are given. Mr. H. truly observes, that the least deviation between the line of pressure and that of the natural course of the canal at the part where the instrument may happen to be, is sure to do harm, as it invariably tends to lay the foundation for the production of false passages; and the case of the physician already quoted, sufficiently proves that no person is equal to the task of introducing a bougie upon himself without the risk of getting into this dilemma; a circumstance that may prove an endless source of embarrassment to the surgeon, when at some future time it may perhaps be essential to the life of the patient, that an instrument should be got into the bladder; since the false passage is sure to catch the point of the catheter or bougie, rendering it next to impossible to introduce either into the receptacle of the urine.

"The most likely means for enabling us to avoid this difficulty, is suggested by what we learn from the morbid anatomy of the parts. We find, almost invariably, that when a false passage is produced in any part beyond the curvature of the urethra, it is on the posterior side of the canal, and consequently if the instrument passed has sufficient firmness to admit of its point being pressed against the opposite or anterior part of the urethra in its way on to the bladder, it may slip past the opening that leads out of the urethra, and by this means be enabled to reach its destination in safety." 204.

The irritable stricture is extremely difficult to manage; if left to itself, it is sure to increase; and if meddled with, in order to its being relieved by the bougie, the attempt only serves to aggravate the disease! The treatment therefore must be left to the discretion of the surgeon, and modulated according to the peculiar state of the parts or patient. It is here, and in the advanced stages of permanent stricture that CAUSTIC has been recommended.

"And where the circumstances of the case are suited to the treatment, it certainly becomes an invaluable remedy; but it is a remedy that sometimes materially involves the future comfort of the patient; on which account, it should never be hastily determined on." p. 206.

The caustic certainly has the effect of diminishing spasm and irritation, and is therefore sometimes the only remedy which we possess for irritable stricture. Mr. Howship prefers the caustic alkali to the lunar caustic, as "the most generally effectual, as well as the most safe in the irritable state of stricture."

When frequent inflammations supervene on spasmodic stricture, it becomes converted gradually into a permanent one. The treatment must vary in proportion as this change takes place. The application of caustic may still be required, and may still prove successful; but the milder, or alkaline caustic will not succeed. The contracted part of the urethra is no longer capable of being relaxed, and the only mode of removing the stricture is by destroying it.

"This forms the fairest case for the lunar caustic, provided the contracted part does not include any considerable extent of the canal of the urethra; and notwithstanding some practitioners have of late thought fit to enter their protest against the use of caustic altogether, it seems to me, that these gentlemen have taken a very superficial view of the subject; otherwise it must have appeared, even to them, that in various instances these diseases are not at all manageable by the bougie alone, although from the satisfactory list of cases brought forward, all ending well, it would seem, that a patient's only anxiety need be excited in the selection of the right surgeon; one who even professes to consider his patient's feelings with so lively a sympathy, as to persuade himself that it is not necessary to give pain, but that the objects of surgery may be obtained just as effectually without any such inconvenience." p. 210.

The nearer the orifice of the urethra the obstruction may be, the more safely may caustic be applied, and *vice versa*. The curvature of the urethra, indeed, and the elasticity of the canal are difficulties in the way even of the simple bougie. The hæmorrhage which sometimes follows the application of caustic is certainly an objection; but if this remedy is only had recourse to in extreme cases, and when other methods have failed, the objection cannot be put in competition with the prospect of relief. Mr. H. thinks, and we quite agree with him, that the caustic in such cases is preferable to cutting down upon, laying open, dissecting out, or perforating with a sharp instrument, the stricture.

"From the length of time sometimes required to enable the surgeon to overcome the obstruction, it becomes necessary to ascertain with accuracy from time to time, the exact progress the caustic has made, in order that it may appear whether it is making its way fairly through the stricture, or whether on the contrary it may not be forming a new passage for itself, leading out from the proper line of the urethra. This information is to be acquired by occasionally passing down a soft bougie, and keeping up a gentle pressure against the stricture for some minutes, until having become warm, it has taken the exact impression or figure of that part of the canal; it is indeed customary with most surgeons, to pass down a plain bougie of as large a size as convenient, previous to each application of the caustic, with a view to clear the canal, and at the same time to determine the exact distance of the stricture from the external orifice of the urethra." p. 214.

*Puncture of the Bladder.*—This is done in three different ways, over the pubis; through the perinæum, and through the rectum. Mr. Howship prefers the latter; and with a short extract relative to this operation we shall close our analysis.

"The trocar need not be larger than that which is employed for hydrocele, although it must be somewhat longer, and should have a gentle even curvature. The form of the instrument should be that of the common trocar with a triangular point, as I have seen that the lancet pointed trocar is sure to divide whatever blood-vessels fall in its way, while the triangular point pushes them aside without further injury.

"As to the position of the patient it is not very material, the operation may be conveniently enough performed as he lies in bed.

"The fore-finger well oiled is to be first passed up into the rectum, and the general degree of fulness of the bladder, as well as the most convenient point above the prostate gland, ascertained. When these circumstances are satisfactorily made out, the trocar pushed nearly but not quite through the canula, is to be gently introduced, and passed up until the extremity of the canula corresponding in situation with the point of the finger already in the rectum, is to be felt against the part where the puncture is to take place. The canula being then in the least degree retracted, while the stilet is pressed forward, places the instrument at once in a fit state for making the puncture; keeping in view the line of direction tending to the centre of the distended bladder; the trocar is now to be steadily passed forward through the coats of the intestine and bladder, when the stilet being carefully withdrawn the canula must be retained, and the urine allowed to pass off.

"As the accidental slipping of the canula out of the orifice in the bladder has been productive of inconvenience from the premature healing of the wound, and the consequent necessity of making a fresh puncture, it is desirable to have a little plate attached to the external part of the canula, perforated with holes, so as to admit of its being secured to a bandage passing round the waist and between the thighs. This precaution, however, generally becomes unnecessary after the first week or two, as the opening usually very soon loses its disposition to heal, until the restoration of the natural passage of the urine renders it useless." p. 217.

A great variety of interesting cases follow, in exemplification of Mr. Howship's general observations; but for which we must refer to the book itself, which will be in the hands of most practical surgeons. We shall introduce a short case, however, the last in the volume, as it illustrates a beautiful plate of diseased bladder. (Plate iii. fig. v.)

"James Killet, a labouring man, had for fifteen years previous to his death, been troubled with stricture in the urethra, brought on in the first instance by virulent gonorrhœa. From that period the medical gentleman who attended him had been occasionally called in to relieve him from the retention of urine; which for the most part was readily accomplished by the introduction of a small sized common bougie; latterly, however, the stricture had become so contracted as to barely admit the smallest catgut bougie that could be procured.

"On these occasions, the instrument was usually suffered to remain some little time, and upon withdrawing it the urine generally flowed. These attacks were commonly preceded by inebriety.

"For some time previous to his death, he complained of considerable pain in passing his urine, as well as difficulty in getting rid of it; the attempt was frequently followed by the appearance of a few drops of blood, and a purulent discharge. The desire to make water proved a constant source of distress, and it came away in very small quantities, almost guttatim.

"When visited in his last attack, every endeavour was made to relieve him by the means formerly made use of; but the introduction of the bougie not succeeding, he was put into the warm bath, in which he voided urine in small quantity but with great pain; to relieve which, opiates were administered.

"The following day the urethra gave way, a quantity of urine escaped into the cellular membrane, the perinæum sloughed, and the urine escaped by the opening; a very few days after this he expired."

"*Examination.*—On examining the body, the bladder was considerably thickened, but not much contracted, for it still contained near a pint of urine. When the parts were removed and laid open, the internal surface of the bladder was found very much diseased. The inner membrane had apparently suffered repeated attacks of violent inflammation: On some parts the natural surface of the membrane was still visible, but rendered of a bright red colour from inflammation; on other parts effused coagulable lymph had become covered with a reddish brown crust of adherent calculous matter; this change had taken place to a considerable extent about the fundus of the bladder, and also in the commencement of the urethra, at the neck of the bladder.

" The prostatal and membranous part of the urethra were much enlarged from the pressure of the urine, as far forward as the stricture, which was at the bulb of the urethra.

" The stricture itself was very nearly impervious, and was of a compact texture, although of no considerable extent.

" When the canal of the urethra had suffered inflammation and distention behind the strictures, coagulable lymph had been effused, and the particles of uric gravel had become adherent through the whole extent of the effused lymph.

" The fistulous orifice where the urethra had burst, was situated immediately behind the strictured part of the canal.

" The prostate gland was considerably enlarged, and had an extensive abscess formed within its substance."

The plates, illustrative of various diseases in the urinary organs, are very beautiful, and inimitably well executed.

In this volume Mr. Howship has not brought forward any new discoveries, or recommended any novel plans of treatment. But he has been very successful in clearly arranging the most accurate descriptions, and urging the most judicious measures for the cure or alleviation of that class of maladies on which he has undertaken to write. In this respect his book forms an excellent reference for the guidance of the junior and less experienced branches of the profession; and to them we most earnestly recommend it as a sound practical treatise on a very interesting and important subject.

